# Anaerobic growth of *Saccharomyces cerevisiae* CEN.PK113-7D does not depend on synthesis or supplementation of unsaturated fatty acids

**DOI:** 10.1093/femsyr/foz060

**Published:** 2019-08-19

**Authors:** Wijb J C Dekker, Sanne J Wiersma, Jonna Bouwknegt, Christiaan Mooiman, Jack T Pronk

**Affiliations:** Delft University of Technology, Department of Biotechnology, Van der Maasweg 9, 2629 HZ Delft, The Netherlands

**Keywords:** *S. cerevisiae*, OLE1, unsaturated fatty acids, anaerobic, oxygen requirement, membrane composition

## Abstract

In *Saccharomyces cerevisiae*, acyl-coenzyme A desaturation by Ole1 requires molecular oxygen. Tween 80, a poly-ethoxylated sorbitan-oleate ester, is therefore routinely included in anaerobic growth media as a source of unsaturated fatty acids (UFAs). During optimization of protocols for anaerobic bioreactor cultivation of this yeast, we consistently observed growth of the laboratory strain *S. cerevisiae* CEN.PK113-7D in media that contained the anaerobic growth factor ergosterol, but lacked UFAs. To minimize oxygen contamination, additional experiments were performed in an anaerobic chamber. After anaerobic precultivation without ergosterol and Tween 80, strain CEN.PK113-7D and a congenic *ole1Δ* strain both grew during three consecutive batch-cultivation cycles on medium that contained ergosterol, but not Tween 80. During these three cycles, no UFAs were detected in biomass of cultures grown without Tween 80, while contents of C10 to C14 saturated fatty acids were higher than in biomass from Tween 80-supplemented cultures. In contrast to its UFA-independent anaerobic growth, aerobic growth of the *ole1Δ* strain strictly depended on Tween 80 supplementation. This study shows that the requirement of anaerobic cultures of *S. cerevisiae* for UFA supplementation is not absolute and provides a basis for further research on the effects of lipid composition on yeast viability and robustness.

## Teaser

*Saccharomyces cerevisiae* CEN.PK113-7D and a congenic *ole1* null mutant grow anaerobically without supplementation of unsaturated fatty acids, which have long been considered as essential anaerobic growth factors for this yeast.

## INTRODUCTION

The large majority of known yeast species ferment glucose to ethanol when grown under oxygen limitation (Barnett, Payne and Yarrow 1979; van Dijken *et al*. 1986). This observation implies that most yeasts do not exclusively depend on mitochondrial respiration for energy metabolism. However, only few yeasts, including *Saccharomyces cerevisiae*, are able to grow on glucose in the complete absence of oxygen (Visser *et al*. 1990; Merico *et al*. 2007). The molecular basis for the non-dissimilatory oxygen requirements of most facultatively fermentative non-*Saccharomyces* yeasts is still not completely understood (Snoek and Steensma 2006; Merico *et al*. 2009).

Anaerobic growth of *S. cerevisiae* imposes special nutritional requirements. Already in the 1950s, Andreasen and Stier (1953a, 1953b) reported that strictly anaerobic growth of *S. cerevisiae* required supplementation of media with a sterol and an unsaturated fatty acid (UFA). Ever since these original observations, synthetic laboratory media for anaerobic growth of *S. cerevisiae* are routinely supplemented with a sterol (usually ergosterol) and a UFA source. The latter is generally provided as Tween 80, a poly-ethoxylated sorbitan ester of oleic acid (Wheeler and Rose 1973; Bulder and Reinink 1974; Fekete, Ganzler and Fekete 2010). While synthesis of nicotinic acid by *S. cerevisiae* is also oxygen dependent (Panozzo *et al*. 2002), this vitamin is not generally considered an anaerobic growth factor, as it is also routinely included in synthetic media for aerobic cultivation of this yeast.

The growth-factor-dependent ability of *Saccharomyces* yeasts to grow anaerobically plays a key role in several of their large-scale industrial applications. In beer fermentation, wort is intensively aerated before inoculation to enable brewing yeast to build up stores of sterols and UFAs for the subsequent anaerobic fermentation process (Casey, Magnus and Ingledew 1983; Depraetere *et al*. 2008). In artisanal wine fermentation, *S. cerevisiae* starts to dominate other ‘wild’ yeast species once oxygen has been depleted during the initial phases of fermentation (Mauricio, Milla and Ortega 1998; Holm Hansen *et al*. 2001).

Sterols and fatty acids are important constituents of cellular membranes. Sterols play a key role in maintenance of membrane integrity and fluidity (Rodriguez *et al*. 1985; Lingwood and Simons 2010), and have also been implicated in specific cellular processes such as endocytosis and nutrient uptake (Umebayashi and Nakano 2003; Pichler and Riezman 2004). The degree of (un)saturation of the fatty-acyl moieties in phospholipids is an important determinant of membrane fluidity (de Kroon, Rijken and de Smet 2013). In addition, fatty acids are involved in energy storage and post-translational modification of proteins (Klug and Daum 2014).

*De novo* biosynthesis of ergosterol, the major sterol in aerobically grown *S. cerevisiae*, involves a monooxygenase (Erg1), demethylase (Erg3), oxidase (Erg25) and desaturases (Erg3 and Erg5) and requires 12 moles of O_2_ per mol of sterol (Summons *et al*. 2006). The oxygen requirement of *S. cerevisiae* for synthesis of UFAs (mainly palmitoleic acid, C16:1, and oleic acid, C18:1, (Martin, Oh and Jiang 2007)) originates from the essential role of the Δ9-fatty acid desaturase Ole1. In the presence of ferrocytochrome b5, Ole1 catalyzes the oxygen-dependent introduction of a *cis* double bond in palmitoyl-CoA and stearoyl-CoA, yielding palmitoleoyl-CoA (C16:1-CoA) and oleoyl-CoA (C18:1-CoA), respectively (Martin, Oh and Jiang 2007; Tehlivets, Scheuringer and Kohlwein 2007). The importance of this reaction is illustrated by the strict UFA auxotrophy of *ole1* null mutants in aerobic cultures (Resnick and Mortimer 1966; Wisnieski and Kiyomoto 1972; Stukey, Mcdonough and Martin 1989).

While no indications for oxygen-independent sterol biosynthesis have been found in nature, neither in living organisms nor in the fossil record (Summons *et al*. 2006), microbial UFA biosynthesis does not universally require oxygen. For example, during acyl-CoA synthesis by bacterial multicomponent type-II fatty-acid synthase (FAS) systems, unsaturated fatty-acyl-CoA intermediates are formed during chain elongation. Following dehydration of the acyl-chain, the double bond of this intermediate of the elongation cycle can be isomerized. This isomerization precludes saturation in subsequent steps and thereby conserves the double bond (White *et al*. 2005). Furthermore, in contrast to the cytosolic *S. cerevisiae* type-I FAS complex that only produces saturated fatty acids (Tehlivets, Scheuringer and Kohlwein 2007), some bacterial type-I FAS proteins are capable of oxygen-independent UFA synthesis (Stuible, Meurer and Schweizer 1997; Radmacher *et al*. 2005).

Based on reported biomass contents, oxygen requirements for UFAs and sterols in *S. cerevisiae* each amount to ∼0.1 mmol O_2_ (g biomass)^−1^ (Bulder and Reinink 1974; Otero *et al*. 2010; Caspeta *et al*. 2014). However, it should be noted that UFA and sterol contents strongly depend on strain background and culture conditions (Deytieux *et al*. 2005). In laboratory-scale cultures, which have a high surface-to-volume ratio, extensive precautions have to be taken to prevent such small amounts of oxygen from entering cultures. For example, cultivation in serum flasks requires removal of oxygen by autoclaving and use of septa that are highly resistant to oxygen diffusion (Miller and Wolin 1974). Minimizing entry of small amounts of oxygen into bench-top laboratory bioreactors is even more challenging and requires use of ultrapure nitrogen gas, applying overpressure and using special materials for tubing and septa (Visser *et al*. 1990; da Costa *et al*. 2018). Furthermore, as indicated by the practice of aerobically ‘loading’ brewing yeasts (Casey *et al*. 1983; Depraetere *et al*. 2008), intracellular stores of ergosterol and UFAs of aerobically pregrown yeast cells may support several generations of growth upon transfer to anaerobic media that lack these anaerobic growth factors.

This paper describes how, during experiments aimed at optimizing bioreactor cultivation protocols for anaerobic growth of the laboratory strain *S. cerevisiae* CEN.PK113-7D (Entian and Kötter 2007; Nijkamp *et al*. 2012), growth was consistently observed in synthetic media that were not supplemented with UFAs, while elimination of both sterols and UFAs almost completely blocked growth. These observations led to the hypothesis that, in contrast to the common assumption in the literature on anaerobic yeast physiology, *S. cerevisiae* does not absolutely require UFAs for anaerobic growth. To test this hypothesis, we analyzed growth of *S. cerevisiae* CEN.PK113-7D and a congenic *ole1* null mutant in cultures grown in an anaerobic chamber and analyzed the lipid composition of anaerobically grown biomass.

## MATERIALS AND METHODS

### Strains, media and maintenance

*S. cerevisiae* strains used and constructed in this study (Table 1) were derived from the CEN.PK lineage (Entian and Kötter 2007; Nijkamp *et al*. 2012). Yeast extract peptone dextrose medium (YPD; 10 g L^−1^ Bacto yeast extract, 20 g L^−1^ Bacto peptone, 20 g L^−1^ glucose) was used for making frozen stock cultures. Synthetic medium with 20 g L^−1^ glucose (SMD) was prepared as described previously (Verduyn *et al*. 1992). Synthetic urea medium (SMD-urea), in which ammonium sulfate was replaced by 2.3 g L^−1^ urea and 6.6 g L^−1^ K_2_SO_4_ was prepared as described earlier (Luttik *et al*. 2000). Similarly, for selection of transformants carrying the amdS marker cassette, ammonium sulfate in SMD was replaced by 10 mM acetamide and 6.6 g L^−1^ K_2_SO_4_ (Solis-Escalante *et al*. 2013). SM media and YP media were autoclaved at 121 and 110°C, respectively, for 20 min. Where indicated, unsaturated fatty acids and/or sterols were added to autoclaved media as Tween 80 (polyethylene glycol sorbate monooleate, Merck, Darmstadt, Germany) and ergosterol (≥95% pure, Sigma-Aldrich, St. Louis, MO), respectively. Concentrated stock solutions of these anaerobic growth factors were prepared by dissolving 8.4 g Tween 80 (equivalent to 7.8 mL) and 0.2 g ergosterol in 17 mL of absolute ethanol, or by dissolving 0.2 g ergosterol in 25 mL absolute ethanol. These stock solutions were incubated at 80°C for 20 min before diluting them 800-fold in growth medium, yielding final concentrations of 420 mg L^−1^ Tween 80 and/or 10 mg L^−1^ ergosterol. *Escherichia coli* XL1-Blue was grown in Lysogeny Broth (LB; 10 g L^−1^ Bacto tryptone, 5 g L^−1^ Bacto yeast extract and 5 g L^−1^ NaCl). For selection of transformants, LB was supplemented with 100 mg L^−1^ ampicillin. After addition of sterile glycerol (30% v/v), culture samples were frozen and stored at −80°C. 'Super optimal broth' (SOB) medium contained 0.5 g L^−1^ yeast extract, 2 g L^−1^ Bacto tryptone, 10 mM NaCl, 2.5 mM KCl, 10 mM MgCl_2_·6H_2_O, 10 mM MgSO_4_·7H_2_O, and was autoclaved at 121°C for 20 min. To prepare 'super optimal broth medium with catabolite repression' (SOC), a concentrated solution of glucose, separately autoclaved at 110° for 30 min, was added to SOB to a final concentration of 20 mM.

**Figure 2.**
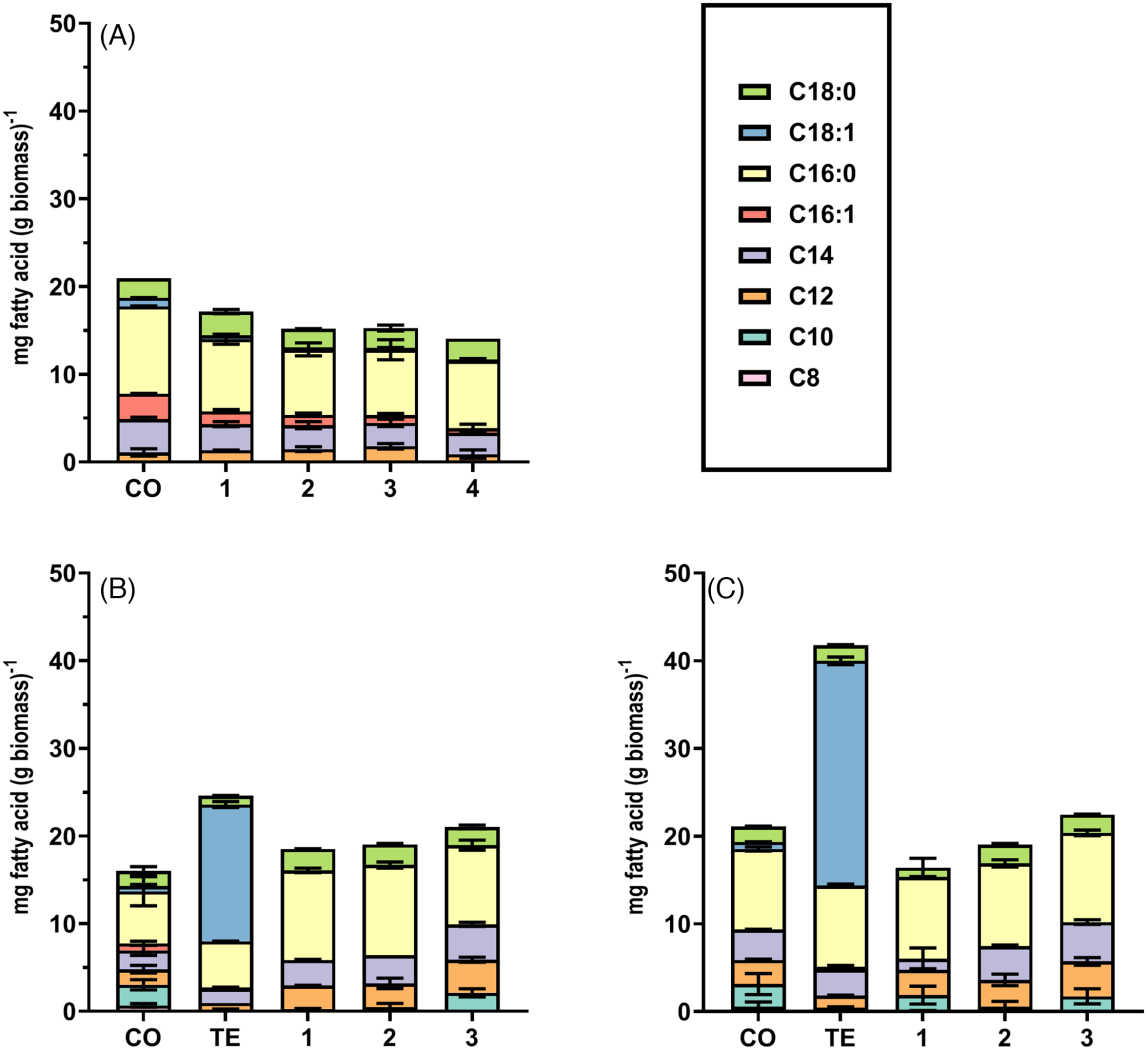
Fatty acid composition of anaerobic *S. cerevisiae* cultures. Fatty acid composition, analyzed by GC-FID, of anaerobically grown cultures in SBRs **(A)** and anaerobic-chamber shake-flask cultures **(B, C)**. (A) Fatty acid composition of the reference strain CEN.PK113–7D during anaerobic SBR cultivation; ‘CO’: carry-over cycle; 1–4: subsequent SBR cycles 1–4 on synthetic medium supplemented with ergosterol but not Tween 80. (B) and (C) Fatty acid composition of strains IMX585 (CEN.PK113–7D with Cas9 integrated in genome) and its congenic ole1Δ mutant IMK861, respectively, during serial-transfer shake-flask cultivation in an anaerobic chamber; ‘CO’: carry-over culture; ‘TE’: cultures grown on synthetic medium supplemented with both anaerobic growth factors; 1–3: transfers 1–3, respectively, of cultures grown on synthetic medium supplemented with ergosterol but not Tween 80. Each panel shows data from independent duplicate bioreactor or shake-flask cultures grown on synthetic medium.

**Table 1.** Strains used in this study.

Name	Relevant genotype	Parental strain	Origin
CEN.PK113-7D	*MATα MAL2*-*8c SUC2 CAN1*	-	Entian and Kötter (2007)
IMX585	*MATα MAL2*-*8c SUC2 can1Δ::cas9-natNT2 URA3 TRP1 LEU2 HIS3*	CEN.PK113-7D	Mans *et al*.(2015)
IMK861	*MATα MAL2*-*8c SUC2 can1Δ::cas9-natNT2 URA3 TRP1 LEU2 HIS3 ole1Δ*	IMX585	This study

### Molecular biology techniques

To amplify DNA fragments for plasmid construction, Phusion® High-Fidelity DNA Polymerase (Thermo Scientific, Waltham, MA) was applied as specified in the manufacturer's protocol, using PAGE-purified oligonucleotide primers (Sigma-Aldrich). Diagnostic polymerase chain reaction (PCR) was performed with DreamTaq PCR Master Mix (Thermo Scientific), according to the manufacturer's protocol and with desalted oligonucleotide primers (Sigma-Aldrich). PCR-amplified linear integration cassettes were purified from 1% (w/v) agarose gels (TopVision Agarose, Thermo Fisher) with TAE buffer (50x, Thermo Fisher) using a Zymoclean Gel DNA Recovery Kit (Zymo Research, Irvine, CA). *E. coli* XL1-Blue competent cells were transformed by heat shock for 40 s at 42°C and, after 1 h recovery at 37°C in SOC medium, plated on selective LB ampicillin media. The GenElute Plasmid Miniprep kit (Thermo Fisher Scientific) was used to isolate plasmids from overnight cultures in 15 mL Greiner tubes on selective medium. *S. cerevisiae* was transformed with the lithium-acetate method (Gietz and Woods 2002). Transformants were selected on SMD agar with acetamide as sole nitrogen source. Single-cell lines of transformants were obtained by three consecutive re-streaks on solid selective medium.

### Plasmid and strain construction

Markerless CRISPR/Cas9-based genome editing of *S. cerevisiae* was performed as described previously (Mans *et al*. 2015). Oligonucleotides and plasmids used in this study are listed in Tables 2 and 3, respectively. A unique guide-RNA (gRNA) sequence targeting *OLE1* was designed using Yeastriction (Mans *et al*. 2015) and synthesized as a 103 bp oligonucleotide (Sigma). To construct the *OLE1*-targeting CRISPR plasmid pUDR319, the plasmid backbone of pROS11 was first PCR-amplified with the double-binding primer 6005. The gRNA-targeting sequence was then introduced as 5′ primer overhang with the double-binding primer 11986, using pROS11 as template. Subsequently, both PCR products were gel purified, digested with *DpnI* (Thermo Scientific) and mixed in equimolar ratio. Gibson assembly was performed in a final volume of 5 µL with the NEBuilder HiFi DNA assembly master mix (NEB, Ipswich, MA), according to manufacturer's instructions. Assembled plasmids were transformed into *E. coli* and selected on solid LB-ampicillin medium. To delete *OLE1* in *S. cerevisiae*, 500 ng of the gRNA plasmid (pUDR319) was transformed to strain IMX585, together with 400 ng of the annealed 120 bp double-strand DNA repair fragment (oligonucleotides 11239 & 11240). This repair fragment consisted of homologous 60 bp sequences immediately up- and downstream of the *OLE1* coding sequence. Cells were selected on solid SM with acetamide as nitrogen source for plasmid selection and Tween 80 to supplement UFA auxotrophic transformants. Deletion of *OLE1* was verified by diagnostic PCR amplification with primers 11249 & 11250. The CRISPR gRNA plasmid was removed by cultivation in YPD with Tween 80 and subsequent single-cell selection on SMD agar plates with Tween 80. Plasmid loss was checked by streaking the resulting single-colony isolates on SMD with 5-fluoroacetamide (Solis-Escalante *et al.*^2013^).

**Table 2.** Primers used in this study.

Purpose	Primer nr.	Sequence 5′→ 3′
gRNA primer targeting *OLE1*	11231	TGCGCATGTTTCGGCGTTCGAAACTTCTCCGCAGTGAAAGATAAATGATCCTTTTGTTCTTGTTGAATCAGTTTTAGAGCTAGAAATAGCAAGTTAAAATAAG
Repair fragment, *OLE1* upper strand	11239	CATAGTAATAGATAGTTGTGGTGATCATATTATAAACAGCACTAAAACATTACAACAAAGGTATCACATTACAATAACAAAACTGCAACTACCATAAAAAAAAATTGAAAAATCATAAAA
Repair fragment, *OLE1* lower strand	11240	TTTTATGATTTTTCAATTTTTTTTTATGGTAGTTGCAGTTTTGTTATTGTAATGTGATACCTTTGTTGTAATGTTTTAGTGCTGTTTATAATATGATCACCACAACTATCTATTACTATG
Diagnostic primer *OLE1* fw	11249	GGTATCCCAGCCTTCTCTGC
Diagnostic primer *OLE1* rv	11250	CTATTGCTCCAGGGCCCAG

**Table 3.** Plasmids used in this study.

Name	Relevant characteristics	Origin
pROS11	2 µm ampR *amdSYM* p*SNR52*-gRNA*_CAN1.Y_*p*SNR52*-gRNA*_ADE.Y_*	Mans *et al*.(2015)
pUDR319	2 µm ampR *amdSYM* p*SNR52*-gRNA*_OLE1_*p*SNR52*-gRNA*_OLE1_*	This study

Note: gRNA target sequences are indicated in subscript.

### Aerobic growth studies in shake flasks

Aerobic growth studies of *S. cerevisiae* strains were performed in 500-mL round-bottom shake flasks filled with 100 mL SMD containing 20 g L^−1^ glucose as carbon source, with or without supplementation of Tween 80. Precultures were inoculated from frozen glycerol stocks and grown overnight on the same medium and used to inoculate fresh flasks, at an initial optical density at 660 nm (OD_660_) of 0.2. OD_660_ was monitored at regular time intervals using a 7200 visible spectrophotometer (Jenway, Staffordshire, UK). All aerobic shake-flask experiments were carried out in duplicate, in an Innova shaker incubator (New Brunswick Scientific, Edison, NJ) set at 30°C and 200 rpm.

### Anaerobic bioreactor cultivation

Anaerobic bioreactor batch cultivation was performed in 2-L laboratory bioreactors (Applikon, Schiedam, the Netherlands) with a working volume of 1.2 L. Before autoclaving, bioreactors were tested for gas leakage by applying 0.3 bar overpressure while completely submerging them in water. Anaerobic conditions were maintained by continuous flushing of the headspace of bioreactor cultures with 500 mL N_2_ min^−1^ (≤0.5 ppm O_2_, HiQ Nitrogen 6.0, Linde Gas Benelux, Schiedam, the Netherlands) and, after inoculation, by maintaining an overpressure of 0.2 bar in the headspace. Oxygen diffusion was minimized by using Fluran tubing (14 Barrer O_2_, F-5500-A, Saint-Gobain, Courbevoie, France) and Viton O-rings (Eriks, Alkmaar, the Netherlands). Furthermore, bioreactor cultures were grown on SMD-urea (Luttik *et al*. 2000) to eliminate the need for pH control and, thereby, to prevent oxygen entry via alkali titration or diffusion through pH probes. The autoclaved mineral salts solution was supplemented with 0.2 g L^−1^ sterile antifoam emulsion C (Sigma-Aldrich, St. Louis, MA). Bioreactors were continuously stirred at 800 rpm and temperature was controlled at 30°C. The outlet gas of bioreactors was cooled to 4°C in a condenser to minimize evaporation of water and volatile metabolites and dried with a PermaPure PD-50T-12MPP dryer (Permapure, Lakewood, NJ) prior to analysis. CO_2_ concentrations in the outlet gas were measured with an NGA 2000 Rosemount gas analyzer (Emerson, St. Louis, MO). The gas analyzer was calibrated with reference gas containing 3.03% CO_2_ and N6 grade N_2_ (Linde Gas Benelux).

Frozen glycerol stock cultures were used to inoculate aerobic 100-mL shake-flask cultures on SMD. After overnight cultivation at 30°C, a second 100-mL aerobic shake-flask preculture on SMD was inoculated at an OD_660_ of 1.0. During the exponential growth phase of this second preculture, biomass was harvested by centrifugation at 4000 *g* for 5 min and washed with sterile demineralized water. The resulting cell suspension was used to inoculate anaerobic bioreactors at an initial OD_660_ of 0.2. No ergosterol or Tween 80 were included in the medium for the first bioreactor batch cultivation cycle (‘carry-over cycle’), in order to deplete endogenous stores of sterols and UFAs.

After the carry-over cycle, cultures were continued in sequential batch reactor (SBR) mode. When the percentage of CO_2_ in the outlet gas dropped sharply to zero, indicating nutrient depletion, a next SBR cycle was manually initiated by removing culture broth with a Masterflex peristaltic pump, until only 25 mL of culture was left in the reactor. The bioreactor was then refilled to 1.2 L with fresh medium with a peristaltic pump and electric level sensor, which corresponded to a 48-fold dilution of the remaining culture sample. The 5-L glass medium reservoir vessel was sparged with N5.5 grade nitrogen gas (Linde Gas Benelux) for at least one h before refilling. Immediately before refilling, ∼20 mL medium was purged from the medium inlet line to remove any oxygen contamination in stagnant medium. To further minimize oxygen contamination, gassing was temporarily switched from headspace to sparging during refilling and overpressure (0.2 bar) was applied throughout empty-refill cycles.

### Anaerobic growth studies in shake flasks

Anaerobic shake-flask experiments were performed in a Shel Lab Bactron 300 anaerobic workstation (Sheldon Manufacturing Inc., Cornelius, OR) at 30°C. The anaerobic gas mixture used for flushing the work space and air lock consisted of 85% N_2_, 10% CO_2_ and 5% H_2_. An IKA® KS 260 Basic orbital shaker platform (Dijkstra Verenigde BV, Lelystad, The Netherlands) placed in the anaerobic chamber was set at 200 rpm. During anaerobic experiments, the air lock was used fewer than three times per week. To minimize oxygen entry during this procedure, a regenerated Pd catalyst for H_2_-dependent oxygen removal was introduced into the chamber whenever the air lock was used. Cultures were grown in 50-mL round-bottom shake flasks containing 40 mL SMD-urea supplemented with either 20 or 50 g L^−1^ glucose. Concentrated solutions of ergosterol and/or Tween 80 were added as indicated. Sterile growth media were preincubated in the anaerobic chamber for at least 48 h prior to inoculation to allow for complete removal of oxygen. Growth experiments in the anaerobic chamber were started by inoculating anaerobic shake flasks, containing SMD-urea without both ergosterol and Tween 80 and containing 50 g L^−1^ glucose, at an initial OD_600_ of 0.2, from an exponentially growing aerobic preculture on SMD. Growth was measured by periodic measurements of the optical density at 600 nm with an Ultrospec® 10 cell density meter (Biochrom, Cambridge, UK) placed inside the anaerobic chamber. To prevent frequent use of the air lock, supplies of cuvettes, pipet tips and demineralized water were all placed inside the anaerobic workspace before the start of growth experiments. When the OD_600_ of the preculture no longer increased, it was used to inoculate anaerobic cultures on SMD-urea with 20 g L^−1^ glucose at an initial OD_600_ of 0.2.

### Analytical methods

Metabolite concentrations in culture supernatants were analyzed by high-performance liquid chromatography (HPLC) as described previously (Verhoeven *et al*. 2017). Biomass dry weight measurements in SBR cultures were performed at the end of each cultivation cycle, using preweighed nitrocellulose filters (0.45 µm, Gelman Laboratory, Ann Arbor, MI). After filtration of 10 or 20 mL culture samples, filters were washed with demineralized water prior to drying in a microwave oven (20 min at 360 W).

Fatty acids in biomass were analyzed as methyl-ester derivatives by gas chromatography with flame-ionization detection (GC-FID). Biomass samples were harvested by centrifuging at least 30 mL of culture broth at 3000 *g* for 5 min. Pellets were washed once with demineralized water and stored at −80°C. Frozen samples were lyophilized overnight in a freeze-dryer (Alpha 1–4 LD Plus, Christ, Osterode am Harz, Germany) and 20 to 30 mg of lyophilized material was weighed into glass methylation tubes (Article no. 10044604, PYREX^TM^ Borosilicate glass, Thermo Fisher Scientific). After adding 2 mL methanol (Honeywell, Mexico City, Mexico), samples were vortexed thoroughly. After addition of 30–100 µL of a 2 mg mL^−1^ internal standard solution of heptadecanoic acid (≥98% pure, Sigma) 2 mL of 3 M methanolic HCl and 2 mL of n-heptane (Sigma) were added. The resulting mixtures were incubated at 80°C for 2 h, while vortexing thoroughly every 15 min, and then rapidly chilled on ice to room temperature. After addition of 2 mL of Milli-Q water (Merck), samples were again vortexed, and centrifuged at 3000 *g* for 5 min to ensure phase separation. The upper heptane phase was transferred to a 2 mL Eppendorf tube (Greiner Bio-One, Alphen aan den Rijn, The Netherlands) containing 10–20 mg dried Na_2_SO_4_ (Merck) to remove remaining traces of water and shaken vigorously. After centrifugation (5 min at 5000 *g*), the liquid phase was transferred to a GC vial (11 mm crimp-neck vial (10326042) and cap (11821653) with butyl rubber septum (Thermo Fisher Scientific)). The sample was concentrated by evaporating the solvent under a stream of N_2_. Fatty acid methyl esters were analyzed on an Agilent Technologies 7890A GC-FID system equipped with an FID-1000–220 Gas Station (Parker Balston, Haverhill, MA, USA) and an Agilent Technologies 7693 Autosampler. A VF-5 ms column (30 m, 0.25 mm internal diameter, 0.25 µm film thickness, Agilent part no. CP9013) was used for separation, and nitrogen was used as a carrier gas at a constant flow of 0.4 mL min^−1^. The oven temperature, which was initially 50°C, was increased to 220°C at 60°C min^−1^, then kept constant for 3 min, increased to 250°C at 8°C min^−1^, again kept constant for 3 min, and finally increased to 320°C at 60°C min^−1^ and kept constant for another 6 min. Inlet temperature was set at 150°C, and FID temperature at 280°C. The Supelco FAME mix C8-C24 (Sigma-Aldrich, MO, USA) was used to calibrate the GC-FID system for quantification of individual fatty acid methyl esters. A separate 10-point calibration curve was made with methyl oleate (>99%, Sigma-Aldrich). Data were adjusted for internal standard concentrations and expressed per g of lyophilized biomass.

## RESULTS

### Minimal growth in anaerobic batch bioreactors of *S. cerevisiae* in the absence of sterols and unsaturated fatty acids

**Figure 1.**
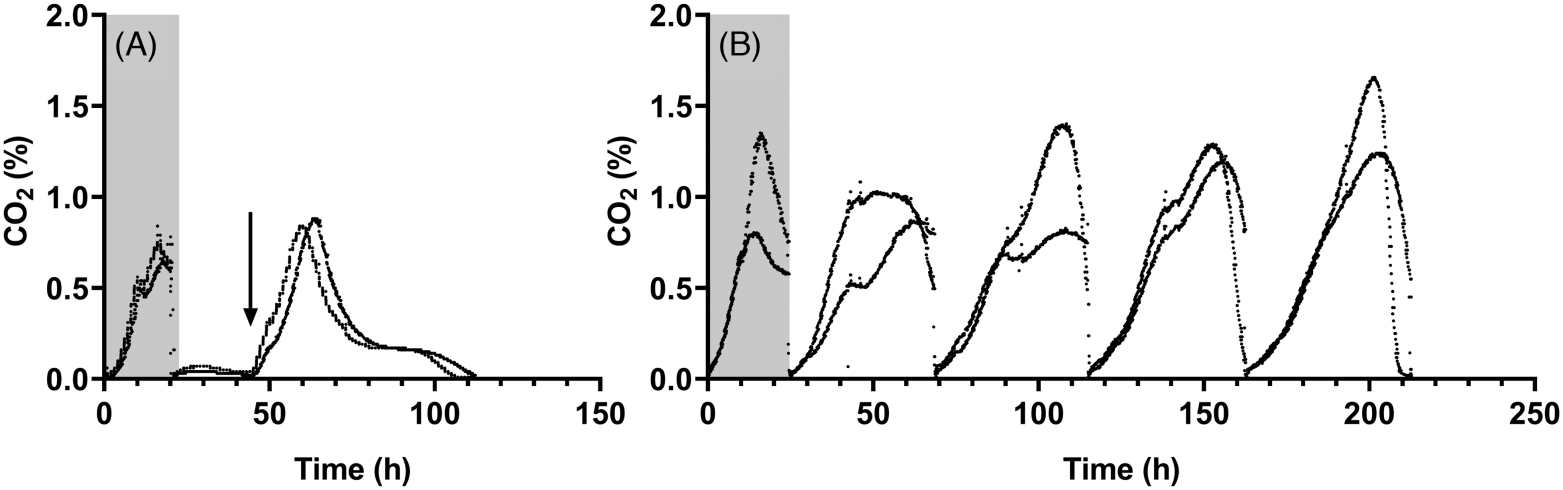
CO_2_ production profiles of anaerobic bioreactor batch cultures of *S. cerevisiae* CEN.PK113–7D. Each panel shows data from independent duplicate bioreactor cultures grown on synthetic medium. Experiments were started with a ‘carry-over’ bioreactor batch culture on synthetic medium without ergosterol and Tween 80 (gray boxes, glucose concentrations in panels A and B were 25 and 40 g L^−1^, respectively). After emptying and refilling with fresh medium, cultures were continued as follows: **(A)** second cycle of growth on synthetic medium with glucose (25 g L^−1^) without ergosterol and Tween 80. An ergosterol pulse (0.55 mg L^−1^) was administered at the time point indicated by the arrow. **(B)** Four SBR cycles on synthetic medium with glucose (40 g L^−1^), supplemented with 10 mg L^−1^ ergosterol but not with Tween 80.

In view of reported technical challenges in achieving strictly anaerobic growth conditions in laboratory bioreactor cultures of yeasts (Visser *et al*. 1990; da Costa *et al*. 2018), we attempted to eliminate sources of oxygen contamination in bioreactor batch cultures of *S. cerevisiae*. The bioreactor headspace was continuously flushed with ultrapure nitrogen gas and kept under overpressure; special tubing and septa were used to minimize oxygen diffusion and no pH or oxygen sensors were used. To assess whether these measures were successful, bioreactor batch cultures were grown on synthetic medium without the anaerobic growth factors ergosterol and Tween 80. After inoculation with an aerobically grown preculture, CO_2_ production rapidly increased until, after 17 h, it reached a maximum and subsequently decreased (Fig. 1A). At this stage, the biomass concentration in the cultures had increased from 0.21 ± 0.00 to 0.60 ± 0.02 g L^−1^ while the glucose concentration was still above 14 g L^−1^ (Figure S1, Supporting Information). Reactors were then emptied, leaving 25 mL culture broth in the reactor, and refilled with fresh medium without ergosterol and Tween 80. In the subsequent batch culture, CO_2_ production was much lower than in the first culture and remained stable for 24 h (Fig. 1A). Optical density measurements showed that fewer than two biomass doublings had occurred over this period, leading to a biomass concentration of ∼0.2 g L^−1^. These results strongly suggested that growth in the first anaerobic batch culture was supported by ‘carry over’ of anaerobic growth factors from aerobically pregrown inoculum. An initial anaerobic cultivation cycle on medium without ergosterol and Tween 80 (‘carry-over culture’) was therefore implemented in all subsequent experiments in anaerobic bioreactors, as well as in growth experiments in an anaerobic chamber.

Although the slow increase of the biomass concentration during the second cultivation cycle suggested that oxygen entry had not been completely eliminated, the experimental set-up was considered suitable for further studies on anaerobic growth-factor requirements. As a pilot experiment, ergosterol (0.55 mg L^−1^) was administered 24 h into the second anaerobic cultivation cycle. After ergosterol addition, CO_2_ production rapidly accelerated and the biomass concentration increased to 0.86 ± 0.03 g L^−1^ (Fig. 1A), indicating that, during the second cultivation cycle, growth in the anaerobic bioreactors was restricted by sterol availability. Since no Tween 80 was added, this observation raised questions about the requirement of these anaerobic cultures for UFAs.

### Omission of UFAs does not prevent growth in anaerobic SBR cultures

To further investigate the observed anaerobic growth of *S. cerevisiae* CEN.PK113-7D in synthetic medium supplemented with ergosterol, but not with Tween 80, experiments were performed in SBRs. After an initial carry-over cycle, four consecutive SBR cycles on medium without Tween 80 showed a pronounced CO_2_ production and corresponding increase of the biomass concentration (Fig. 1B). Specific growth rates estimated from the exponential phases of CO_2_ production, as well as estimated biomass yields on glucose, were similar throughout these four cycles (Table 4). To investigate whether growth without Tween 80 supplementation reflected *de novo* UFA biosynthesis, enabled by inadvertent entry of oxygen into the bioreactors, fatty acids were extracted from biomass harvested at the end of each SBR cycle and analyzed by gas chromatography. At the end of the ‘carry-over’ cycle, but also at the end of the subsequent four cycles on medium from which Tween 80 was omitted, small quantities of palmitoleate (C16:1) and oleate (C18:1) were detected (Fig. 2A; Table S2, Supporting Information). Since the four SBR cycles led to an ∼5·10^6^-fold dilution of any UFAs remaining in yeast biomass after the initial carry-over cycle, presence of these UFAs most probably indicated *de novo* UFA synthesis due to leakage of oxygen into the reactors.

**Table 4.** Anaerobic growth of *S. cerevisiae* CEN.PK113-7D in anaerobic SBR cultures on synthetic medium with glucose, supplemented with ergosterol (10 mg·L^−1^), but without UFA supplementation. In the initial carry-over cycle (CO, see Fig. 1B), also ergosterol was omitted. Specific growth rates were estimated from CO_2_ production profiles. Yields of ethanol and biomass were estimated from measurements of biomass, glucose and ethanol at the start and end of each SBR cycle. Data are represented as mean and standard error of the mean of data from independent duplicate cultures.

Cycle	Biomass (g L^−1^)	µ (h^−1^)	Y_Ethanol/glucose_ (g g^−1^)	Y_Biomass/glucose_ (g g^−1^)
CO	0.84 ± 0.09	0.42 ± 0.00	0.37 ± 0.01	0.05 ± 0.00
1	1.30 ± 0.06	0.20 ± 0.02	0.38 ± 0.00	0.04 ± 0.00
2	1.48 ± 0.16	0.17 ± 0.01	0.38 ± 0.02	0.04 ± 0.00
3	1.55 ± 0.02	0.17 ± 0.00	0.37 ± 0.00	0.04 ± 0.00
4	1.57 ± 0.04	0.14 ± 0.02	0.37 ± 0.01	0.04 ± 0.00

### UFA-independent growth of a reference strain and an *ole1* null mutant in an anaerobic chamber

**Figure 3.**
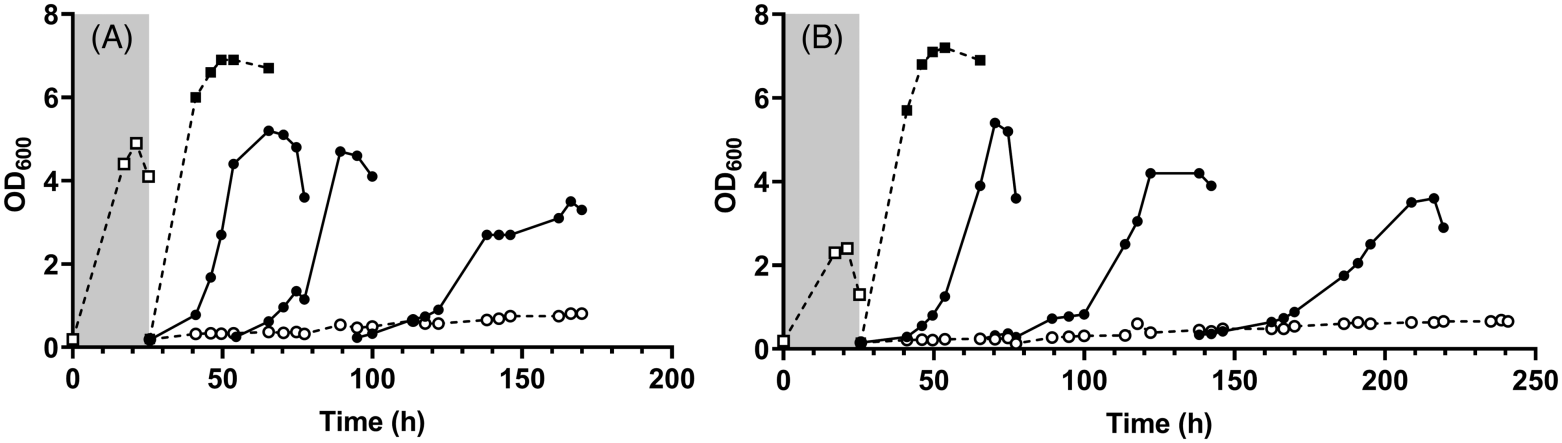
UFA-independent anaerobic growth of the reference strain *S. cerevisiae* IMX585 and the ole1Δ strain IMK861. Strains IMX585 **(A)** and IMK861 (ole1Δ) **(B)** were grown in shake-flask cultures placed in an anaerobic chamber. Aerobic precultures were used to inoculate an anaerobic preculture (‘carry-over culture’, open squares, gray box) on SMD containing 50 g L^−1^ glucose but no anaerobic growth factors. When the OD_600_ no longer increased, cultures were transferred to fresh SMD, supplemented either with Tween 80 and ergosterol (closed squares), only ergosterol (closed circles) or neither (open circles). The initial culture to which only ergosterol was added (closed circles, first line) was sequentially transferred to the same medium (closed circles, second and third line). The data are from a single representative experiment of biological duplicate cultures. Data of the duplicate experiment are shown in Figure S2 (Supporting Information).

To further reduce oxygen contamination, UFA-independent anaerobic growth of the reference strain *S. cerevisiae* IMX585 (CEN.PK113-7D with a chromosomally integrated Cas9 expression cassette, Mans *et al*. 2015) was studied in an anaerobic chamber equipped with a H_2_/Pd catalyst system to scavenge traces of oxygen. Since *ole1Δ* strains of *S. cerevisiae* are unable to synthesize UFAs (Resnick and Mortimer 1966; Stukey, Mcdonough and Martin 1989), growth of the congenic *ole1Δ* strain IMK861 was studied in the same system to exclude the possibility of *de novo* UFA synthesis. As observed in SBR cultures, both strains grew during an initial anaerobic ‘carry-over’ culture on medium without sterols or UFAs. However, upon transfer to a second anaerobic shake-flask culture without these supplements, virtually no growth was observed for the two strains over a period of 180 h (Fig. 3).

After the initial carry-over culture, both the reference strain and the *ole1Δ* mutant grew to similar optical densities in medium supplemented with both Tween 80 and ergosterol, indicating that deletion of *OLE1* did not negatively affect growth in UFA-supplemented anaerobic cultures. In addition, both strains grew in three consecutive transfers in anaerobic shake flasks containing synthetic medium supplemented with only ergosterol. During these serial transfers, similar maximum optical densities were again reached for both strains (Fig. 3). These observations suggested that, at least in the CEN.PK genetic background, synthesis or supplementation of UFAs is not required for anaerobic growth of *S. cerevisiae*. This hypothesis was further tested by analyzing the lipid content and composition of yeast biomass in the serial transfer experiments.

At the end of the carry-over cultures, UFAs were detected in both strains (Fig. 2B and C). Since growth in the carry-over cultures ceased before glucose was depleted (Table S1, Supporting Information), this observation suggested that depletion of sterols rather than depletion of UFAs caused growth to stop. No UFAs were detected during three subsequent transfers in medium without Tween 80, neither in the reference strain nor in the *ole1Δ* mutant. Instead, contents of palmitic acid (C16:0) and short-chain saturated fatty acids (C10-C14) were higher than in cultures supplemented with Tween 80 (Fig. 2B and C; Table S2, Supporting Information).

When cells from a stationary-phase carry-over culture were instead transferred to medium containing both ergosterol and Tween 80, oleic acid (C18:1), which is the main UFA side-chain of Tween 80 (Bulder and Reinink 1974), was the dominant fatty acid in yeast biomass (Fig. 2B and C; Table S2, Supporting Information).

### UFA synthesis or supplementation is essential for aerobic growth

**Figure 4.**
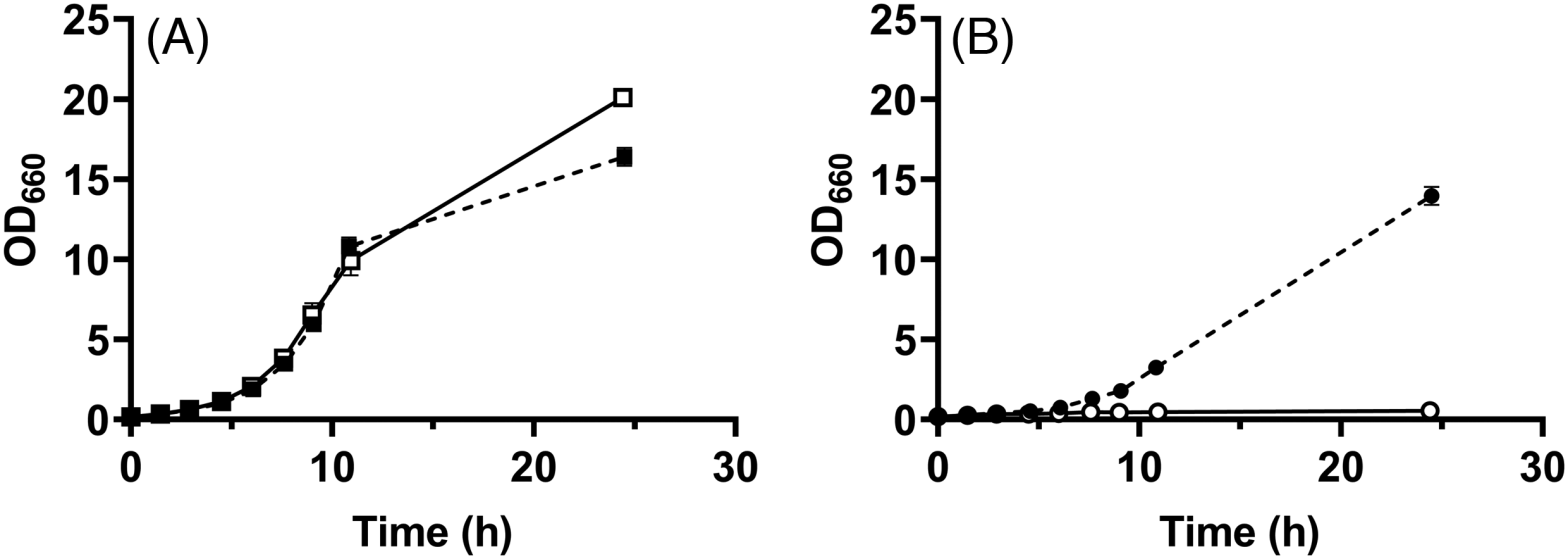
Aerobic growth of *S. cerevisiae* strains IMX585 and IMK861 (ole1Δ) in the presence and absence of a source of unsaturated fatty acids. Strains IMX585 **(A)** and IMK861 (ole1Δ) **(B)** were grown in 100 mL SMD in 500 mL round-bottom shake-flasks at 30°C and at 200 rpm. Growth was monitored in SMD supplemented with Tween 80 (closed symbols) and in SMD without Tween 80 (open symbols). Data represent mean and standard error of the mean of independent biological duplicate cultures.

Several previous studies reported that *ole1Δ* strains constructed in other *S. cerevisiae* genetic backgrounds are unable to grow aerobically without UFA supplementation (Resnick and Mortimer 1966; Stukey, Mcdonough and Martin 1989; Giaever *et al*. 2002). To check if an *ole1* null mutation in the CEN.PK genetic background might have a different phenotype, we investigated aerobic growth of the *ole1Δ* strain IMK861 in shake-flask cultures. These experiments confirmed that, also in the CEN.PK genetic background, aerobic growth on a glucose synthetic medium strictly depended on UFA supplementation after transfer from an aerobic Tween 80-supplemented preculture (Fig. 4).

## DISCUSSION

For over six decades, yeast researchers have based the design of anaerobic growth media on the assumption that anaerobic growth of *S. cerevisiae* strictly requires UFA supplementation. While this study confirms previous reports that synthesis or supplementation of UFAs is required for aerobic growth of *S. cerevisiae* (Stukey, Mcdonough and Martin 1989; Giaever *et al*. 2002), it indicates that, surprisingly, the UFA requirement for anaerobic growth of *S. cerevisiae* is not absolute.

Since nonrespiratory oxygen requirements of *S. cerevisiae* are small (Rosenfeld and Beauvoit 2003), interpretation of results can easily be obscured by oxygen contamination and by ‘carry-over’ of anaerobic growth factors from aerobic or growth-factor-supplemented precultures. Indeed, oxygen contamination of bioreactor experiments was evident from synthesis of small amounts of palmitoleic and oleic acid (Fig. 2A; Table S1, Supporting Information). This UFA synthesis occurred despite extensive precautions to prevent oxygen entry, which sufficed to severely restrict growth in the absence of both ergosterol and Tween 80. Residual production of unsaturated fatty acids, despite extensive measures to exclude oxygen, was also observed in a recent chemostat study in which both ergosterol and Tween 80 were omitted from growth media (da Costa *et al*. 2019). No K_m_ values for oxygen of *S. cerevisiae* Ole1 or related Δ9 desaturases have been reported in the literature. However, these results suggest that Ole1 has a very high affinity for oxygen which, even under extreme oxygen limitation, enables yeast cells to efficiently scavenge oxygen for UFA synthesis.

Serial-transfer experiments in an anaerobic chamber, equipped with a Pd/H_2_ system to remove traces of oxygen, did not show detectable UFA levels in biomass grown on synthetic medium without Tween 80. Nevertheless, after an initial ‘carry-over’ culture, growth of a reference strain and of an *ole1Δ* mutant continued during three consecutive transfers in UFA-free medium. UFA contents were already below detection limit after the first cycle and, after the second cycle, biomass of the carry-over culture had been diluted by ∼500-fold. Although these results do not entirely exclude a minute UFA requirement for anaerobic growth, any remaining UFA levels in the serial batch cultures were too low to account for maintenance of membrane fluidity (Degreif *et al*. 2017). Anaerobic cultures in UFA-free medium showed increased contents of medium-chain (C10 to C14) fatty acids. This adaptation is in line with the demonstrated flexibility of the yeast lipidome in response to other environmental stresses (Okuyama *et al*. 1979; Klose *et al*. 2012). A similar adaptation was previously observed in promitochondria of cells after anaerobic incubation without a source of UFAs (Paltauf and Schatz 1969) and in a recent chemostat study on severe oxygen limitation in chemostat cultures without UFA or sterol supplementation (da Costa *et al*.^2019^).

Anaerobic growth of *S. cerevisiae* is only rarely studied in media that contain sterols, but not UFAs. In the original work of Andreasen and Stier, cell counts that reached in cultures that were supplemented with only ergosterol were slightly higher than in controls with only UFA supplementation or in the absence of both growth factors (Andreasen and Stier 1953b). One reason for the routine inclusion of Tween 80 is that this surfactant aids distribution of highly hydrophobic sterols in aqueous media (Bikhazi and Higuchi 1971). A requirement of anaerobic *S. cerevisiae* cultures for UFA supplementation is often inferred from the well-documented UFA auxotrophy of *ole1* null mutants in aerobic cultures. Stukey, Mcdonough and Martin (1989) showed that aerobic growth of an *ole1* null mutant ceased when the contribution of UFAs decreased below 7.3% mol of the total fatty acid content. The sustained anaerobic growth of an *ole1Δ* mutant in UFA-free media, with undetectable intracellular UFA contents (Figs 2 and 3), reveals that UFA requirements of *S. cerevisiae* strongly depend on oxygen status.

While experimentally addressing the question why UFA requirements of aerobic and anaerobic *S. cerevisiae* cultures differ is beyond the scope of this study, at least two hypotheses can be formulated based on the literature. Esterification of sterols with fatty acids, predominantly with oleate (C18:1-Δ9), plays a key role in the complex regulation of sterol homeostasis (Ferreira *et al*. 2004) and steryl-ester synthesis has been demonstrated to decrease during anaerobiosis (Valachovič, Hronská and Hapala 2001). The lower sterol content of anaerobic *S. cerevisiae* cultures may well render them less sensitive to UFA depletion. In mammalian cells, oleate prevents mitochondrial generation of reactive oxygen species (ROS) under palmitate stress (Yuzefovych, Wilson and Rachek 2010). If the same mechanism occurs in yeast mitochondria, absence of respiratory ROS generation in anaerobic cultures could offer an explanation for their tolerance to UFA depletion. Shifting anaerobically grown, UFA-free cultures of an *ole1Δ* mutant to aerobic conditions should provide a relevant experimental system to further explore this interesting problem.

Although our results indicate that *S. cerevisiae* CEN.PK113-7D does not absolutely require UFAs during anaerobic growth, elimination of Tween 80 from growth media negatively affected growth rate and biomass yield. SBR cultures supplemented with ergosterol but not Tween 80, in which the biomass still contained small amounts of palmitoleic and oleic acid (Table S2, Supporting Information), showed an estimated specific growth rate of 0.14 and 0.20 h^−1^ (Table 4). This value is significantly lower than reported for anaerobic batch cultures of *S. cerevisiae* CEN.PK113-7D supplemented with both Tween 80 and ergosterol (Bisschops *et al*. 2015). Biomass yields on glucose (0.04 g biomass (g glucose)^−1^, Table 4) were ∼2-fold lower than in anaerobic chemostat cultures grown with Tween 80 supplementation (Boender *et al*. 2009). This low biomass yield might reflect increased leakage of protons and/or other solutes across UFA-depleted membranes, for example caused by mislocalization of proteins in membranes with a high proportion of saturated lipids (Budin *et al*. 2018). Increased membrane permeability may also contribute to the lag phases observed in anaerobic chamber experiments upon transfer of stationary-phase cultures, grown without Tween 80, to fresh UFA-free medium (Fig. 3).

We hope that our results, which were generated with yeast strains belonging to a single genetic background and under a limited set of experimental conditions, will inspire further research into the physiology and ecological relevance of UFA-independent growth of yeasts. As illustrated by the strain and context dependency of lipid composition in aerobic *S. cerevisiae* cultures (Daum *et al*. 1999; Otero *et al*. 2010; Madsen *et al*. 2011), it is relevant to explore whether UFA-independent growth also occurs in other *S. cerevisiae* genetic backgrounds and in related species and genera. Further research is also needed to investigate the impact of UFA depletion on robustness of *S. cerevisiae* cultures, for example, at low pH and at different temperatures. Furthermore, availability of a *S. cerevisiae* strain that can grow without UFA supplementation provides an interesting starting point for laboratory evolution experiments and studies on membrane engineering for improved cellular performance (Degreif *et al*. 2017; Ma *et al*. 2019).

## FUNDING

This work was funded by an Advanced Grant of the European Research Council to JTP (grant #694633).

## Supplementary Material

foz060_Supplemental_Files
