# Supplementary material for: Anaerobic growth of Saccharomyces cerevisiae CEN.PK113-7D does not depend on synthesis or supplementation of unsaturated fatty acids
Source: FEMS Yeast Res. 2019 Aug 19;19(6):foz060. doi: 10.1093/femsyr/foz060 (PMC6750169; doi:10.1093/femsyr/foz060)
Supplement: foz060_Supplemental_Files [file foz060_supplemental_files.zip › Dekker_and_Wiersma_2019_supplementary.docx]

# Supplementary data


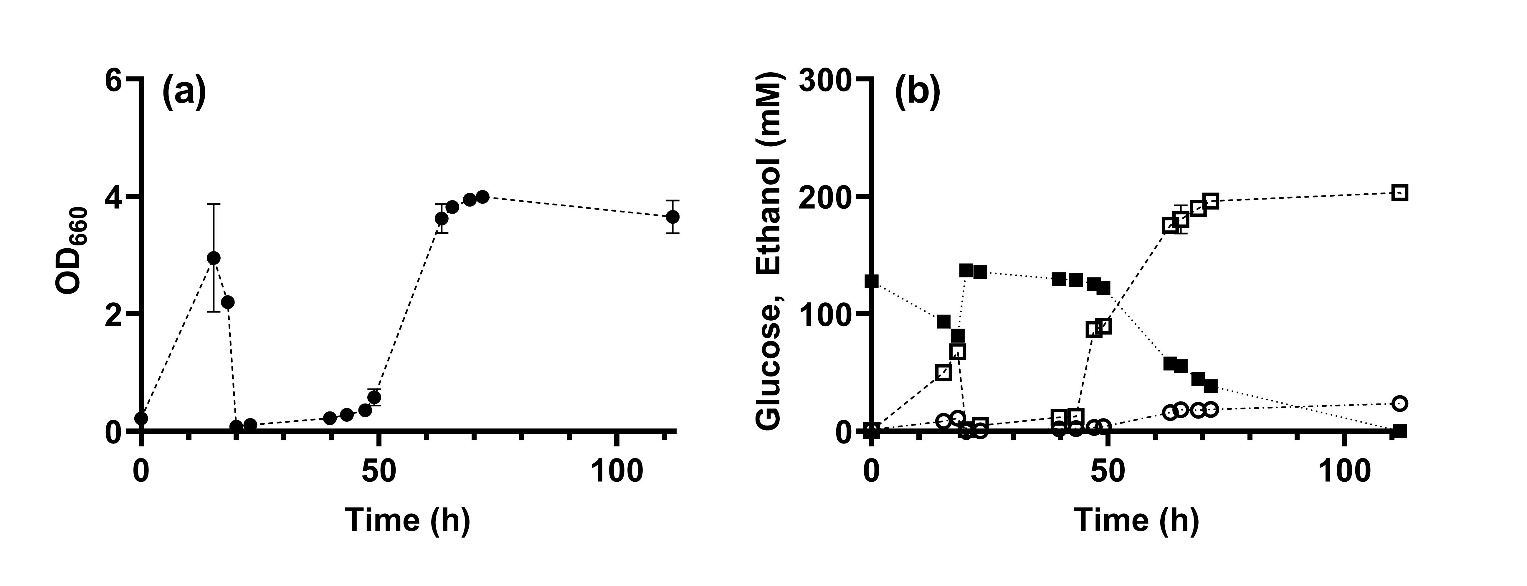


**Figure S1: Anaerobic growth profiles of CEN.PK113-7D in bioreactor cultures on SMD-urea.** Carry-over bioreactor cultures on synthetic medium with 25 g L^-1^ glucose and, initially, no Tween 80 or ergosterol, were inoculated from aerobic shake-flask cultures. Upon observation of a decrease in optical density a new batch cycle was initiated by emptying and refilling with new media. Subsequently, at approximately 48 h, a pulse of ergosterol dissolved in ethanol (0.55 mg L^-1^) was added. (A) optical density measurements, (B) metabolite profiles of glucose (closed squares), ethanol (open squares) and glycerol (open circles) over time. Data represent mean and standard error of the mean from two independent biological cultures.


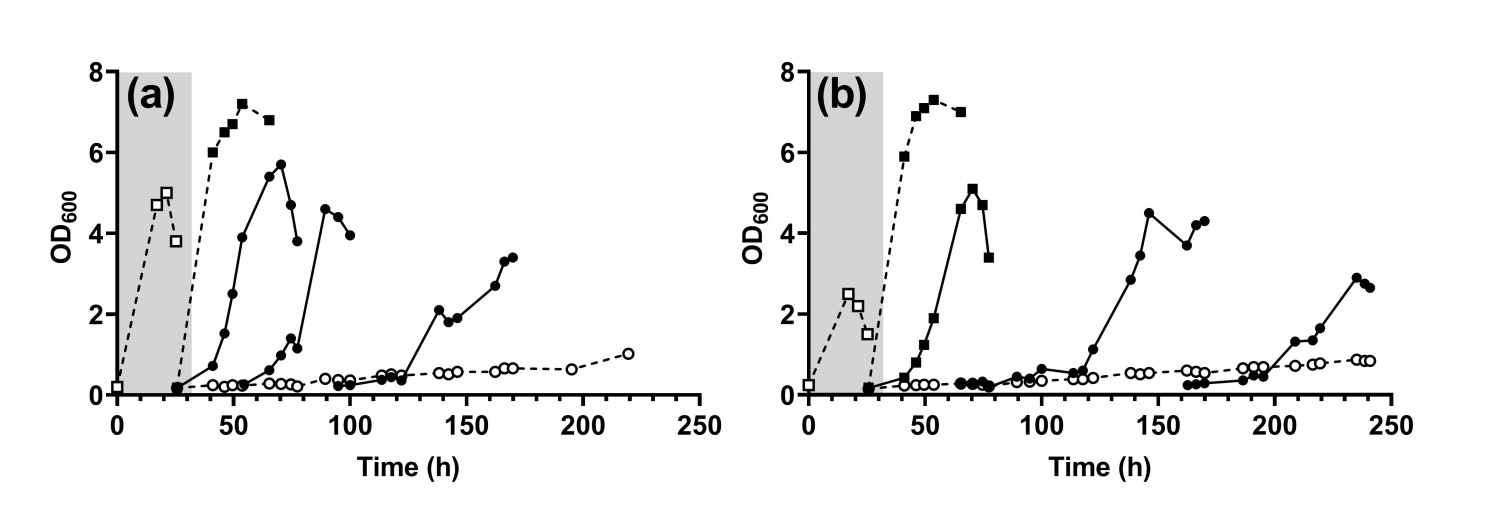


**Figure S2: Biological duplicates of anaerobic growth experiment in anaerobic chamber of the reference strain *S. cerevisiae* IMX585 and the *ole1****Δ* **strain IMK861.** Strains IMX585 (A) and IMK861 (*ole1Δ*, B) were grown on glucose synthetic medium in shake-flask cultures placed in an anaerobic chamber. Aerobic pre-cultures were used to inoculate an anaerobic pre-culture (‘carry-over culture’, open squares, grey box) on synthetic medium containing 50 g L^-1^ glucose but no anaerobic growth factors. When the OD_600_ no longer increased, cultures were transferred to fresh synthetic medium with 20 g L^-1^ glucose and supplemented with either both anaerobic growth factors (Tween 80 and ergosterol, closed squares), only ergosterol (closed circles), or neither of the growth factors (open circles). The initial culture to which only ergosterol was added (closed circles, first line) was sequentially transferred to medium of the same composition (closed circles, second and third lines). The data are of one representative experiment of biological duplicate cultures.

**Table S1: Residual glucose of shake-flask cultures in anaerobic chamber upon termination of the experiment.** Residual glucose as measured by HPLC upon termination of the anaerobic shake-flask experiment. ‘CO’ = carry-over batch, ‘TE’ = culture supplemented with Tween 80 and ergosterol, 1-3 = subsequent cycles of growth on synthetic medium supplemented with only ergosterol. Data represent mean and standard error of the mean of independent biological duplicate cultures.

| **Cycle** | **Residual glucose (mM)** | |
| --- | --- | --- |
|  | IMX585 | IMK861 (*ole1Δ*) |
| CO | 91.6 ± 0.8 | 200.0 ± 0.6 |
| TE | 0 | 0 |
| 1 | 0 | 0 |
| 2 | 0 | 0 |
| 3 | 0 | 0 |

**Table S2: Fatty acid composition of biomass of anaerobic yeast cultures.** Data represent mean and standard error of the mean of two biological duplicate cultures. ‘CO’ = carry-over batch, ‘TE’ = culture supplemented with Tween 80 and ergosterol, 1-4 = subsequent cycles of growth on medium supplemented with only ergosterol. Data corresponds to Figure 2.

| **Fatty acid composition in mg fatty acid (g biomass)^-1^** | | | | | | | | | |
| --- | --- | --- | --- | --- | --- | --- | --- | --- | --- |
|  | **C8** | **C10** | **C12** | **C14** | **C16:1** | **C16:0** | **C18:1** | **C18:0** | **Total** |
| ***Anaerobic SBR, CEN.PK113-7D*** | | | | | | | | | |
| CO | - | - | 1.10 ±0.42 | 3.81 ±0.21 | 2.90 ±0.02 | 9.94 ±0.05 | 0.98 ±0.03 | 2.22 ±0.01 | 20.93 ±0.73 |
| 1 | - | - | 1.34 ±0.02 | 2.98 ±0.28 | 1.48 ±0.20 | 8.21 ±0.58 | 0.44 ±0.11 | 2.71 ±0.25 | 17.15 ±0.33 |
| 2 | - | - | 1.48 ±0.28 | 2.73 ±0.40 | 1.18 ±0.19 | 7.45 ±0.74 | 0.25 ±0.01 | 2.12 ±0.02 | 15.20 ±1.59 |
| 3 | - | - | 1.80 ±0.31 | 2.67 ±0.42 | 0.89 ±0.17 | 7.45 ±1.15 | 0.22 ±0.03 | 2.26 ±0.34 | 15.28 ±2.01 |
| 4 | - | - | 0.90 ±0.48 | 2.43 ±0.15 | 0.53 ±0.45 | 7.70 ±0.00 | 0.16 ±0.10 | 2.37 ±0.00 | 14.07 ±0.08 |
| ***Anaerobic shake-flasks, IMX585*** | | | | | | | | | |
| CO | 0.68 ±0.20 | 2.36 ±0.59 | 1.78 ±0.44 | 2.15 ±0.58 | 0.81 ±0.24 | 5.94 ±1.66 | 0.61 ±0.17 | 1.70 ±0.49 | 16.01 ±4.35 |
| TE | - | 0.20 ±0.08 | 0.77 ±0.01 | 1.65 ±0.02 | 0.15 ±0.02 | 5.24 ±0.02 | 15.61 ±0.34 | 1.01 ±0.02 | 24.61 ±0.32 |
| 1 | - | 0.27 ±0.05 | 2.67 ±0.04 | 2.91 ±0.07 | - | 10.23 ±0.25 | - | 2.45 ±0.03 | 18.52 ±0.45 |
| 2 | - | 0.45 ±0.45 | 2.74 ±0.60 | 3.20 ±0.01 | - | 10.31 ±0.36 | - | 2.36 ±0.11 | 19.05 ±1.51 |
| 3 | - | 2.11 ±0.47 | 3.78 ±0.29 | 4.04 ±0.23 | - | 9.03 ±0.57 | - | 2.08 ±0.21 | 21.03 ±1.77 |
| ***Anaerobic shake-flasks, IMK861 (ole1Δ)*** | | | | | | | | | |
| CO | 0.55 ±0.55 | 2.58 ±1.19 | 2.74 ±0.12 | 3.49 ±0.04 | - | 9.19 ±0.23 | 0.81 ±0.02 | 1.75 ±0.04 | 21.10 ±1.53 |
| TE | - | 0.45 ±0.10 | 1.39 ±0.03 | 2.93 ±0.02 | 0.36 ±0.13 | 9.24 ±0.16 | 25.63 ±0.43 | 1.78 ±0.08 | 41.77 ±0.69 |
| 1 | 0.07 ±0.05 | 1.80 ±1.02 | 2.89 ±0.12 | 1.25 ±1.25 | - | 9.33 ±0.04 | - | 1.09 ±1.06 | 16.42 ±3.45 |
| 2 | - | 0.58 ±0.58 | 3.03 ±0.66 | 3.86 ±0.13 | - | 9.43 ±0.42 | - | 2.15 ±0.12 | 19.04 ±0.57 |
| 3 | - | 1.74 ±0.86 | 3.99 ±0.44 | 4.48 ±0.26 | - | 10.18 ±0.32 | - | 2.08 ±0.06 | 22.46 ±1.93 |
