# Supplementary figures and images for: Anaerobic growth of Saccharomyces cerevisiae CEN.PK113-7D does not depend on synthesis or supplementation of unsaturated fatty acids
Source: FEMS Yeast Res. 2019 Aug 19;19(6):foz060. doi: 10.1093/femsyr/foz060 (PMC6750169; doi:10.1093/femsyr/foz060)

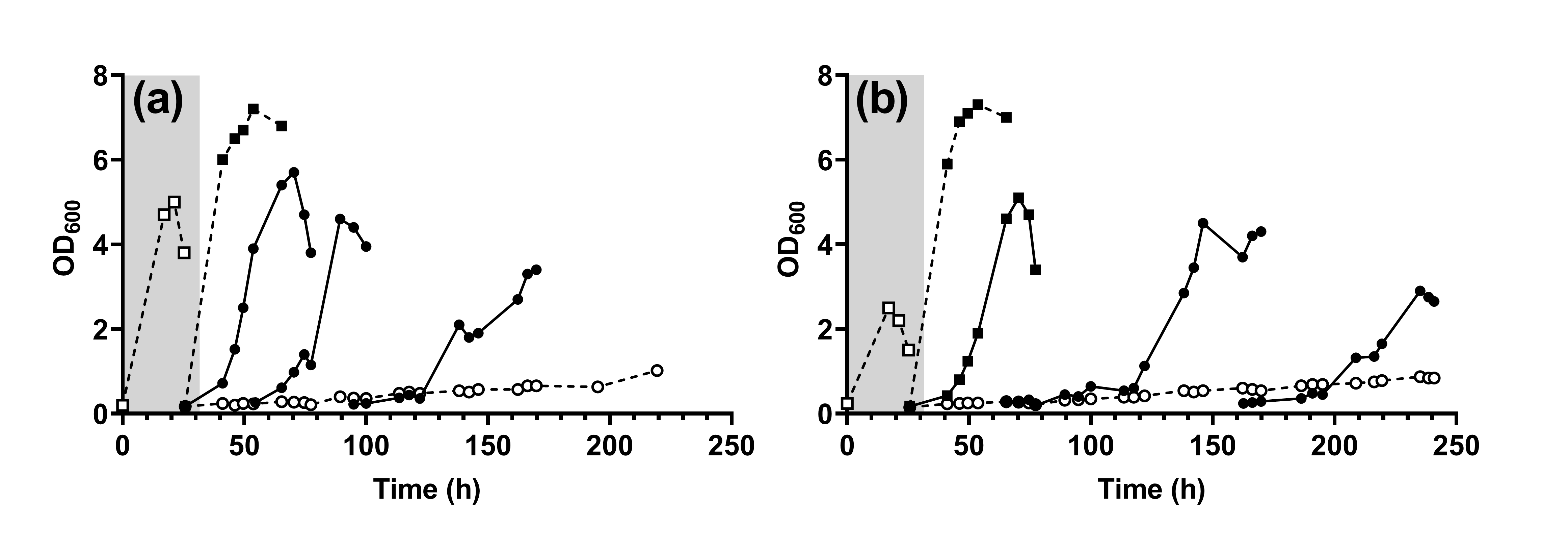

Supplement: foz060_Supplemental_Files [file foz060_supplemental_files.zip › Figure_S2.tif]

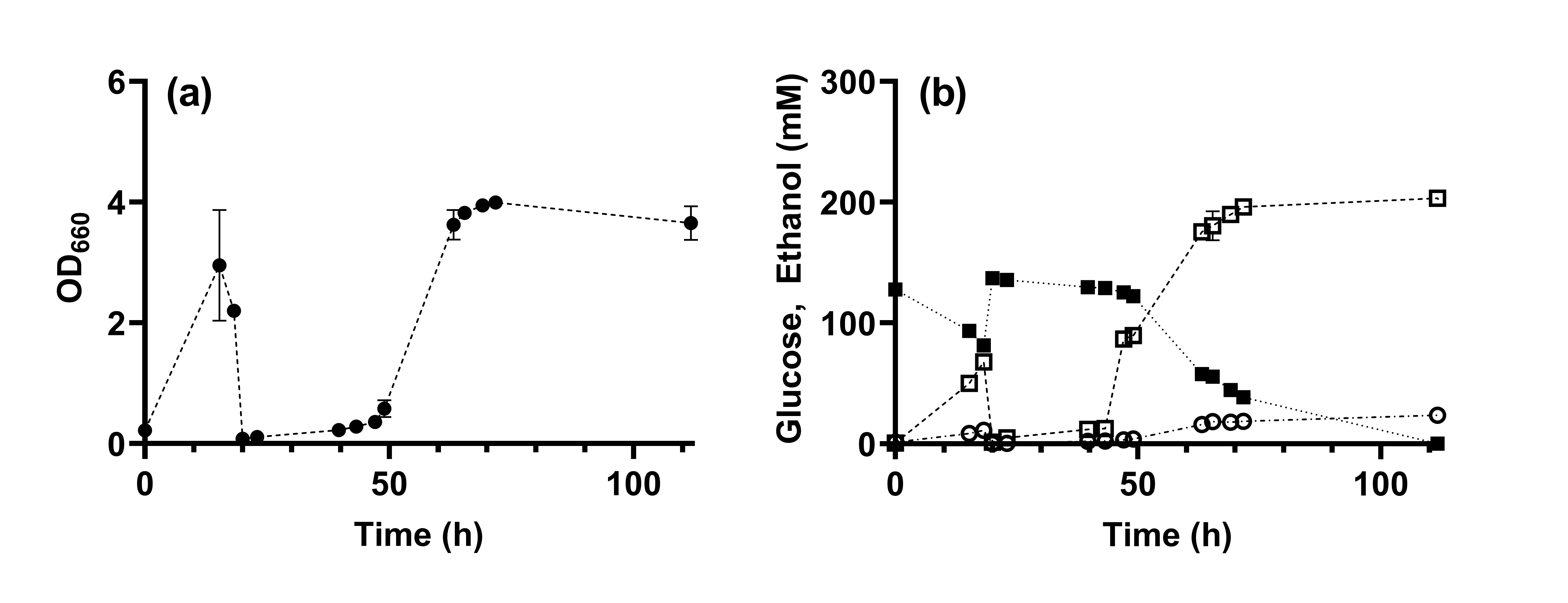

Supplement: foz060_Supplemental_Files [file foz060_supplemental_files.zip › Figure_S1.tif]
